# A Manual of Medical Jurisprudence

**Published:** 1898-03

**Authors:** 


A Manual of Medical Jurisprudence. By Alfred S. Taylor, M. D., Lecturer on Medical Jurisprudence and Chemistry in Guy's Hospital, London. New American edition of 1897 from the twelfth English edition. Thoroughly revised by Clark Bell, Esq., of the New York Bar. In one octavo volume of 831 pages, with fifty-four engravings and eight full-page plates. Philadelphia and New York: Lea Brothers & Co., Publishers. 1897.

Taylor's Medical Jurisprudence has always been a favorite in America. This popularity has been well earned and is due to the careful selection of cases cited. The present edition is a revision of all the previous editions and includes much new material. We hoped to find some reference to the defense of crime by the entering of the plea of hypnotic influence. This subject has more than once been brought up in American courts and its legal status should be defined in all books on medical jurisprudence. In the trial of Gabrielle Bompart, in France, her attorney attempted to prove that she was an accomplice in the murder while under the hypnotic influence of her confederate. That this testimony was not admitted by the court is a fact of so much importance that we regret its omission or any reference to it. Another omission which we regret is any reference to the X-ray. It is important that its use in the courts up to date should be clearly stated. Aside from these two criticisms, we have nothing but praise to say of the work. Its treatment of all subjects is clear and satisfactory. Illustrations are used wherever it is necessary to make clear a certain point, as for instance in the chapter on animal and human red corpuscles. We bespeak for Taylor's Jurisprudence a continuance of the popular favor it has so long enjoyed and merited. J. W. P.

